# Epileptogenic zone characteristics determine effectiveness of electrical transcranial stimulation in epilepsy treatment

**DOI:** 10.1093/braincomms/fcaf012

**Published:** 2025-02-19

**Authors:** Maëva Daoud, Samuel Medina-Villalon, Elodie Garnier, Ionuț-Flavius Bratu, Giada Damiani, Ricardo Salvador, Fabrice Wendling, Giulio Ruffini, Christian Bénar, Francesca Pizzo, Fabrice Bartolomei

**Affiliations:** Epileptology Department and Institut de Neurosciences des Systèmes, INSERM/AMU, 13005 Marseille, France; APHM, Timone Hospital, Epileptology and Cerebral Rhythmology, 13005 Marseille, France; Epileptology Department and Institut de Neurosciences des Systèmes, INSERM/AMU, 13005 Marseille, France; Epileptology Department and Institut de Neurosciences des Systèmes, INSERM/AMU, 13005 Marseille, France; Neuroelectrics, Barcelona, Spain; Neuroelectrics, Barcelona, Spain; Rennes Univ, INSERM, LTSI U1099, F 35000, Rennes, France; Neuroelectrics, Barcelona, Spain; Epileptology Department and Institut de Neurosciences des Systèmes, INSERM/AMU, 13005 Marseille, France; APHM, Timone Hospital, Epileptology and Cerebral Rhythmology, 13005 Marseille, France; APHM, Timone Hospital, Epileptology and Cerebral Rhythmology, 13005 Marseille, France

**Keywords:** multichannel tDCS, SEEG, epileptogenic zone network, treatment responsiveness, electric field modeling

## Abstract

Transcranial direct current stimulation shows promise as a non-invasive therapeutic method for patients with focal drug-resistant epilepsy. However, there is considerable variability in individual responses to transcranial direct current stimulation, and the factors influencing treatment effectiveness in targeted regions are not well understood. We aimed to assess how the extent and depth of the epileptogenic zone and associated networks impact patient responses to transcranial direct current stimulation therapy. We conducted a retrospective analysis of stereoelectroencephalography data from 23 patients participating in a personalized multichannel transcranial direct current stimulation protocol. We evaluated the extent and depth of the epileptogenic zone network, propagation zone network, and the combined network of the entire epileptogenic and propagation zones, correlating these factors with clinical response measured by the reduction in seizure frequency following repeated transcranial direct current stimulation sessions. Among the patients, 10 (43.5%) were classified as responders (R), experiencing a significant (>50%) decrease in seizure frequency, while 13 were non-responders, showing minimal improvement or increased seizure frequency. Importantly, we found a significant positive correlation between the extent of the epileptogenic zone network and changes in seizure frequency. A smaller epileptogenic zone network extent was associated with better transcranial direct current stimulation efficacy, with responders demonstrating a significantly smaller epileptogenic and propagation zones compared with non-responders. Additionally, non-responders tended to have a significantly deeper epileptogenic zone network compared with responders. Our results highlight the significant impact of the extent and depth of the epileptogenic zone network on transcranial direct current stimulation efficacy in patients with refractory focal epilepsy. Responders typically exhibited a smaller and shallower epileptogenic zone network compared with non-responders. These findings suggest that utilizing individualized epileptogenic zone network characteristics could help refine patient selection for personalized transcranial direct current stimulation protocols, potentially improving therapeutic outcomes.

## Teaser

Daoud *et al*. report that transcranial direct current stimulation (tDCS) efficacy in focal drug-resistant epilepsy is influenced by the extent and depth of the epileptogenic zone network. Patients with smaller and shallower epileptogenic zone networks responded better to tDCS, emphasizing the importance of these factors in refining patient selection for future tDCS trials

## Graphical Abstract

**Graphical Abstract:**
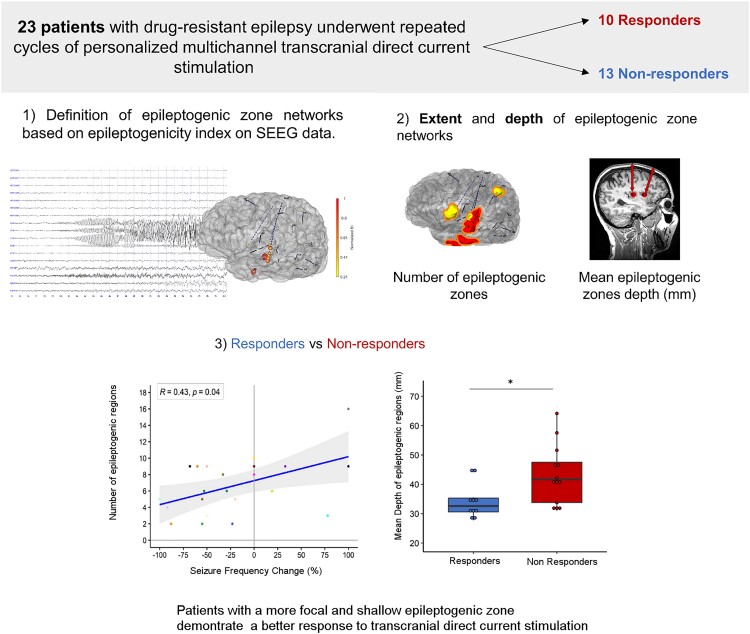

## Introduction

Epilepsy is a neurological condition with high prevalence, affecting over 50 million people worldwide. Despite the effectiveness of pharmacological treatments in controlling seizures for a portion of patients with epilepsy, one-third of patients still experience uncontrolled seizures.^1^ For patients who have focal drug-resistant epilepsy, the surgical resection of the epileptogenic zone appears as a promising strategy to achieve lasting seizure control. However, this approach may not always be indicated and is associated with a non-negligible rate of failures,^2^ leading to the search for alternative treatment solutions. Methods employing non-invasive brain stimulation to modulate brain excitability, such as transcranial direct current stimulation (tDCS), are promising therapeutic options.^3^ This neuromodulation technique delivers a weak current (1–2 mA) through the cortex using scalp electrodes.^4,5^ It has been established that tDCS modulates neural activity through subthreshold membrane depolarization or hyperpolarization. This modulation occurs through the electric field generated by currents between the electrodes, which induce excitation or inhibition depending on field distribution relative the cortical surface.^6-8^ In epilepsy, precise targeting of the cathodal electrode on the epileptogenic zone is crucial for inducing cortical inhibition and alleviating the exacerbated neural activity that characterizes this pathological brain region.

Several controlled trials utilizing tDCS demonstrated favorable clinical outcomes in reducing seizure frequency in adult and paediatric patients with drug-resistant epilepsy.^9-12^ While tDCS is increasingly used for focal refractory epilepsy and has proven to be efficient in some patients, a substantial number of individuals with drug-resistant epilepsy do not respond positively to this treatment. Some studies have documented negative outcomes, without change in epileptic discharges nor improvement in seizure frequency after cathodal tDCS.^13,14^ Fifty to sixty percent of patients included in recent studies, including multichannel tDCS, are not responders.^5,11,15,16^ Moreover, some patients may even experience a transient worsening of seizures after repeated tDCS sessions.^15-17^ Considering the substantial risks associated with seizures, including increased mortality rates, injuries, and the development of related comorbidities, it is essential to address these challenges for non-responder patients. Efforts should focus on mitigating the induced worsening of seizure occurrence and investigating the factors underlying the variable response to tDCS treatment among patients.

Notably, the variability in clinical outcome may arise from the heterogeneity in stimulation parameters, including electrode placement, electrode size, current intensity, stimulation duration, and number of tDCS sessions^18^ but also from patient heterogeneity (etiology, location, and the extent of the epileptogenic zone). The main challenge of tDCS lies in the ability of low-intensity current to penetrate tissues and reach deep brain regions. Indeed, several studies have highlighted the reduction of electric field magnitude due to the depth between scalp stimulation electrodes and the anatomical target.^19-22^ Using multichannel tDCS, incorporating smaller electrode pairs and contrasting with conventional tDCS using two large rectangular electrodes, potentially enhances targeting precision while potentially reaching brain regions.^8,23^ Incorporating multiple pairs of cathodal and anodal electrodes raises concerns about achieving precise targeting of the epileptogenic zone with an inhibitory electric field while minimizing unintended excitation of non-target areas.

In current epilepsy research, it is widely recognized that focal drug-resistant epilepsy should be considered a large-scale brain network disorder, including both the epileptogenic zone network (EZN) and the propagation zone network (PZN).^24-28^ These networks are defined using advanced quantification methods, including the epileptogenicity index (EI), which assesses seizure onset using frequency or time–frequency analysis of intracerebral recordings.^24,29^ This distinction, based on empirical measurements and quantifications, has received a significant degree of validation by being integrated into computational models of digital brain twins.^30,31^ The EZN is a network of brain regions with the highest epileptogenicity, responsible for initiating seizures, often characterized by fast rhythmic discharges and specific seizure onset pattern. The PZN is built of brain regions with lower epileptogenicity, involved in the spread of seizures, typically showing slower and less intense discharges and unable to spontaneously generate seizures.

The specific brain regions involved in the epileptogenic zone EZN are identified through pre-surgical assessment using stereoelectroencephalography (SEEG) recordings, and these regions differ among patients.

To our knowledge, no study has explored whether the EZN characteristics in terms of depth and extent impact the outcome of tDCS. Therefore, our study aimed to retrospectively examine how the characteristics of the epileptogenic network may influence the clinical response observed after multiple multichannel tDCS cycles in patients with focal drug-resistant epilepsy.

## Materials and methods

### Patients and data collection

Between 2019 and 2023, 23 patients who have focal drug-resistant epilepsy participated in a clinical trial based on a personalized multichannel tDCS protocol. Based on inclusion criteria, patients included were at least 12 years of age, had drug-resistant focal epilepsy that was inoperable or had failed prior epilepsy surgery, and had at least four seizures per month at the start of the study. All were required to have undergone an SEEG before inclusion to define a unifocal epileptogenic zone and to be taking stable epilepsy medication throughout the duration of the study. The protocol proceeded as follows: after a 2-month baseline period, each patient received daily stimulation with an individualized multichannel tDCS treatment for 5 consecutive days, constituting a tDCS cycle, as described in study by Daoud *et al*.^16^ This cycle was repeated three times every 2 months for all patients. Each daily treatment consisted of two 20-min stimulation sessions separated by a 20-min break for 40 min of stimulation. The average total injected current was approximately 2 mA. For each patient, personalized electrode positions were determined using an optimization pipeline based on individual factors and biophysical head models derived from patient T1-weighted MRIs.^32^ The Stimweaver algorithm^8^ produced the montage using a target E-field map on the patient’s neocortical surface, considering the regions to be inhibited based on SEEG recordings and identifying ‘silent areas’ without epileptogenic activity. Electrode positions were based on the 10–20 EEG system, typically with cathodes over the regions to inhibit and anodes over silent areas. The summary of stimulation electrodes per patient and the delivered intensities are provided in the Supplementary Table 1.

**Figure 1:**
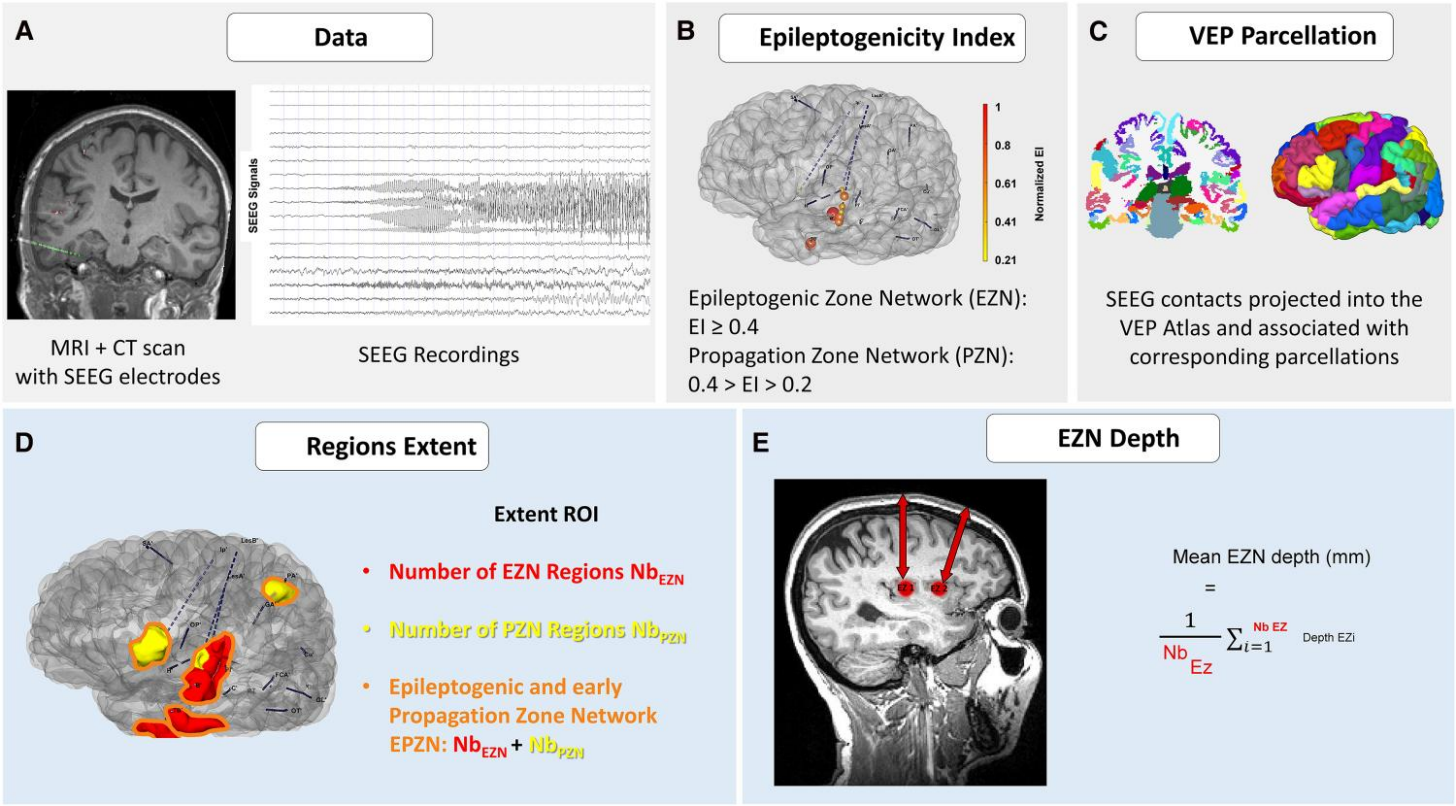
**Overview of the data analysis pipeline.** For the presurgical assessment, each patient underwent SEEG well before the tDCS protocol. **(A)** The co-registration between the CT scan with SEEG electrodes and the patient’s MRI was performed. **(B)** The epileptogenicity index (EI) was calculated from SEEG signals. SEEG contacts were labeled as belonging to the epileptogenic zone (EZN) if EI ≥ 0.4 and as part of the propagation zone (PZN) if 0.4 > EI > 0.2. **(C)** Then SEEG contacts were assigned to anatomical locations within the Virtual Epileptic Patient (VEP) atlas. **(D)** The extent of EZN, PZN and Epileptogenic early propagation zone network EPZN (EZ + PZ) was determined by counting the number of brain regions within these areas for each patient. **(E)** EZN depth was determined by averaging the distance from the scalp to all centroids of epileptogenic regions (EZi). Abbreviations: SEEG: StereoElectroEncephalography; EI: Epileptogenicity Index; EZN: epileptogenic zone network; PZN: propagation zone network; EPZN: epileptogenic and propagation zones networks; Nb: number; ROI: regions of interest, EZi: centroid of the epileptogenic regions, EZ: epileptogenic regions.

For this retrospective study, we collected pre- and post-surgical implantation data, including MRI and CT scans with SEEG electrodes. We used GARDEL (a Graphical User Interface for Automatic Registration and Depth Electrodes Localization), our in-house Matlab-based software (available at https://meg.univ-amu.fr/doku.php?id=epitools:gardel). This tool performed the co-registration of MRI with CT scans, automating the segmentation and precise localization of intracranial electrode contacts using image processing techniques.^33^ GARDEL accurately identifies the position of each SEEG contact within the grey and white matter and assigns its anatomical location using a chosen atlas. Our study used the Virtual Epileptic Patient atlas (VEP Atlas)^34^ (Fig. 1). The VEP atlas subdivides brain regions from Destrieux atlas^35^ to better match clinical practice.

Clinical data were collected by extracting seizure counts from patients’ seizure diaries. We categorized patients into two groups based on their response to the tDCS protocol. The responder group (R) consisted of patients who achieved a reduction of at least 50% in their seizure frequency at a 2-month follow-up after the third tDCS cycle compared with baseline. In contrast, the non-responder group (NR) comprised patients who showed less improvement, no change, or an increase in seizure frequency.

### Determining the extent of the epileptogenic zone and networks

The SEEG analysis used a bipolar montage in the open-source AnyWave software.^36^ An expert clinician (F.P.) identified epileptogenic regions through a combination of visual and quantitative analysis of SEEG signals, mainly using the ‘Epileptogenicity Index’ (EI) method.^33^ This index evaluates the epileptogenicity of brain structures recorded with depth electrodes, considering spectral aspects (e.g. occurrence of fast oscillations replacing background activity) and temporal properties (e.g. delay of appearance with respect to seizure onset time) within intracerebral electroencephalography (EEG) signals.^29^ Elevated EI values indicate structures involved early in the ictal process, generating rapid discharges at seizure onset. Normalized EI values ranging from 0 (indicating no epileptogenicity) to 1 (representing maximal epileptogenicity) were assigned to each brain region of interest, defined based on the VEP atlas.^34^

The concept of three networks—EZN, propagation zone (PZN), and non-involved zones (NIZ)—has been proposed in previous reviews.^24,27^ The use of thresholded EI values to distinguish between these networks has been introduced in previous studies.^37,38^

Two clinical experts (F.P., I.F.B.) classified these regions as part of the EZN or part of the PZN. Regions with an EI ≥ 0.4 were considered EZN regions. In contrast, regions with 0.2 < EI < 0.4 and sustained discharge during seizures were designated as PZN (Fig. 1). If two or more bipolar contacts sampled a region, the maximum EI value obtained for that region was used. EZN and PZN regions defined for each patient are detailed in Supplementary Table 2.

The extent of EZN and PZN was determined by counting the number of brain regions within these areas for each patient. A higher count of EZN regions (with a normalized EI of 0.4 or higher) indicates a more extensive EZN. The entire pathologic network is denoted as the epileptogenic and early propagation networks (EPZN). The EPZN extent was determined by summing the EZN and PZN regions (Fig. 1).

The number of EZN, PZN, and the total number of implanted regions for each patient are reported in Supplementary Table 4

### Assessment of EZ depth

To assess the depth of epileptogenic regions for each patient, we concentrated on EZN regions (with EI ≥ 0.4) due to their close spatial proximity. Subsequently, the anatomical position of each VEP region on the patient’s MRI was projected into a normalized MNI space for analysis. The MRI coordinates of the centroids were acquired using an in-house Matlab script (Mathworks, Naticks, MA, USA). Using the centroid coordinates, we calculated the Euclidean distances in millimetres between the centroid of each region and the skull surface, retaining the minimal distance. Ultimately, to establish the depth of EZN, we averaged the distance from the surface across all centroids of regions within the EZN (Fig. 1).

### Statistical analysis

We investigated differences in epileptogenic region characteristics between responder (R) and NR groups, focusing on their extent and depth. We evaluated the significance of differences in the number of EZN, PZN, and EPZN (EZ + PZ) regions between R and NR groups. Additionally, we compared the mean depth of EZN for R and NR using a Wilcoxon test, with a correction for false discovery rate applied to *P*-values. *P*-values below 0.05 after false discovery rate correction were considered statistically significant.

The second analysis was based on the Spearman correlation coefficient computed between the change in seizure frequency (SF) after tDCS cycles and the number of EZN, PZN, and EPZN regions. Additionally, we examined the correlation between SF change and the mean depth of epileptogenic zone regions. Statistical analyses were performed using RStudio (version 4.2.2), considering a *P*-value of <0.05 (after correction) as statistically significant.

## Results

Table 1 provides clinical information for the 23 included patients. Due to a substantial increase (100%) in their SF after the second tDCS cycle compared with the baseline, two patients (patients 21 and 22) underwent only two cycles (2 weeks) of tDCS instead of the initially planned three cycles. This event led to their exclusion from the last stimulation cycle based on safety protocol guidelines, categorizing them as non-responder patients. Nonetheless, their SF levels returned to baseline after 2 months of follow-up.

**Table 1:** Demographic data and epilepsy characteristics of the patients

Patients	Gender	Age	SZs type	Etiology	Number SEEG electrodes	Number EZ regions	Number PZ regions	ASM	tDCS target	Response
Pat 1	M	20	Focal sensitivity, Visual hallucination	Cryptogenic	9	9	2	3	Left temporo-sylvian, mesio- occipital	NR
Pat 2	F	23	Visual impairment	Heterotopia	10	2	6	3	Left occipital plus posterior baso-temporal	R
Pat 3	M	23	Focal non motor	FCD	9	10	5	2	Left temporo- insular	NR
Pat 4	M	31	Simple focal without loss of awareness, complex focal seizure with loss of awareness, FBTCS	Cryptogenic	16	2	2	2	Left mesio-lateral, temporal	NR
Pat 5	M	41	Focal non motor, partial impaired awareness	Cryptogenic	16	9	4	2	Left temporo opercular	R
Pat 6	F	50	Complex focal, impaired awareness, FBTCS	FCD 1	13	9	3	5	Right pericentral/parietal and posterior temporal	NR
Pat 7	M	42	Complex focal, impaired awareness	Cavernoma	9	3	2	4	Left	NR
									Mesio-lateral, Temporo-orbitofrontal	
Pat 8	M	60	Focal non motor, impaired awareness	Infectious	15	8	5	3	Left temporo basal	NR
Pat 9	M	32	EPC, focal motor seizures	Gliosis	4	6	3	4	Right pericental extended	NR
Pat 10	M	29	Focal motor, impaired awareness	Pre-natal stroke	12	9	1	4	Left fronto parietal sup	R
Pat 11	F	43	Focal, impaired awareness	Schizencephalia, heterotopia, polymicrogyria	19	6	3	3	Left mesio-lateral, temporo-parietal	NR
Pat 12	M	14	Focal motor	Cryptogenic	10	4	1	4	Right paracentral	R
Pat 13	F	16	Complex focal with loss of awareness, FBTCS	FCD 1	13	6	3	3	Bilat pericentral (R > L)	R
Pat 14	F	12	Focal motor, EPC	Cryptogenic	8	2	6	2	Left paracentral	R
Pat 15	M	16	Simple focal, without loss of awareness, paresthesia	Infectious/trauma, MRI of left hemispheric atrophy	15	5	2	4	Left dorso-lateral pre-frontal	R
Pat 16	F	23	Focal sensitive motor without loss of awareness	Cryptogenic	13	3	5	2	Right pericentral	R
Pat 17	F	29	Focal non motor	FCD	13	9	5	3	Left temporal	NR
Pat 18	F	12	EPC, focal motor, generalized seizures	Cryptogenic	8	5	1	3	Left pericentral	R
Pat 19	F	30	Complex focal with loss of awareness, FBTCS	FCD 2	9	8	1	4	Left pericentral	NR
Pat 20	M	48	Complex focal, impaired awareness	Cryptogenic	13	5	6	4	Left mesio-lateral, temporal	NR
Pat 21	F	15	Focal motor without loss of awareness, Paresthesia	Cryptogenic	15	9	5	2	Right perisylvian and post central	NR
Pat 22	M	29	Simple focal without loss of awareness, complex focal with loss of awareness, FBTCS	Cryptogenic	15	16	2	3	Left mesio-lateral, Temporal	NR
									Plus dorso-lateral frontal	
Pat 23	F	31	Focal motor	FCD 2	9	9	1	1	Left pericentral	R

Abbreviations: FCD, focal cortical dysplasia; FCD I, type 1 focal cortical dysplasia; R, responder; NR, non-responder; FCD 2, type 2 focal cortical dysplasia; FBTCS, focal to bilateral tonic-clonic seizures.

**Figure 2:**
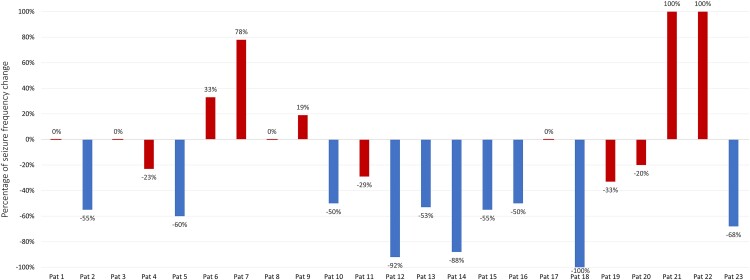
**Percentage change in seizure frequency compared with baseline 2 months after multichannel tDCS cycles (*n* = 23 patients).** The responder patients are represented in blue and the non-responder patients are in red.

The clinical results (Fig. 2) illustrate the SF change 2 months following the last tDCS cycle compared with each patient’s baseline. Specifically, 10 patients were classified as responder patients (R), demonstrating a SF decrease exceeding 50% (mean change −67% ± 19%). The remaining 13 patients, who exhibited either an SF decrease of <50% or an increase compared with baseline, were classified as NR patients (mean change 17% ± 47%). The baseline seizure counts for all patients are presented in Supplementary Table 3.

### Effect of epileptogenic regions’ extent

We investigated how the extent of epileptogenic areas, as determined by the epileptogenicity index, might affect the clinical response to tDCS. We estimated this extent by counting the brain regions involved in epileptogenic regions (EZN, PZN, and EPZN) and targeted by inhibitory stimulation.

**Figure 3:**
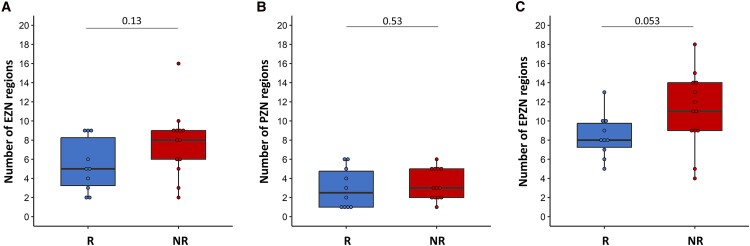
**Comparing the extent of epileptogenic regions in responder (R) And non-responder (NR) patients. (A)** Boxplots illustrate EZN extent determined by the number of EZN regions for R (in blue) and NR (in red) groups (respectively *n* = 10 and *n* = 13). **(B)** For PZN regions. **(C)** For epileptogenic and early propagation network regions (EPZN) (EZN + PZN). Each data point corresponds to the number of the specific brain regions considered for each patient (EZN, PZN and both). The black bar and black point represent the difference (but not significant *pval* = 0.053) between R and NR groups using the Wilcoxon test. ‘*ns*’ refers to a non-significant difference between the groups.

Comparing R and NR groups, no significant difference was observed in the number of EZN regions (*W* = 40.5, *pval* = 0.13) or in the number of PZN regions (*W* = 54.5, *pval* = 0.53; Fig. 3A and B). However, when considering the total extent of the EPZN, calculated by combining EZN and PZN values, we observed a trend indicating a greater extent of the whole epileptogenic and early propagation network in NR patients compared with R (*W* = 54.5, *pval* = 0.053; Fig. 3C).

**Figure 4:**
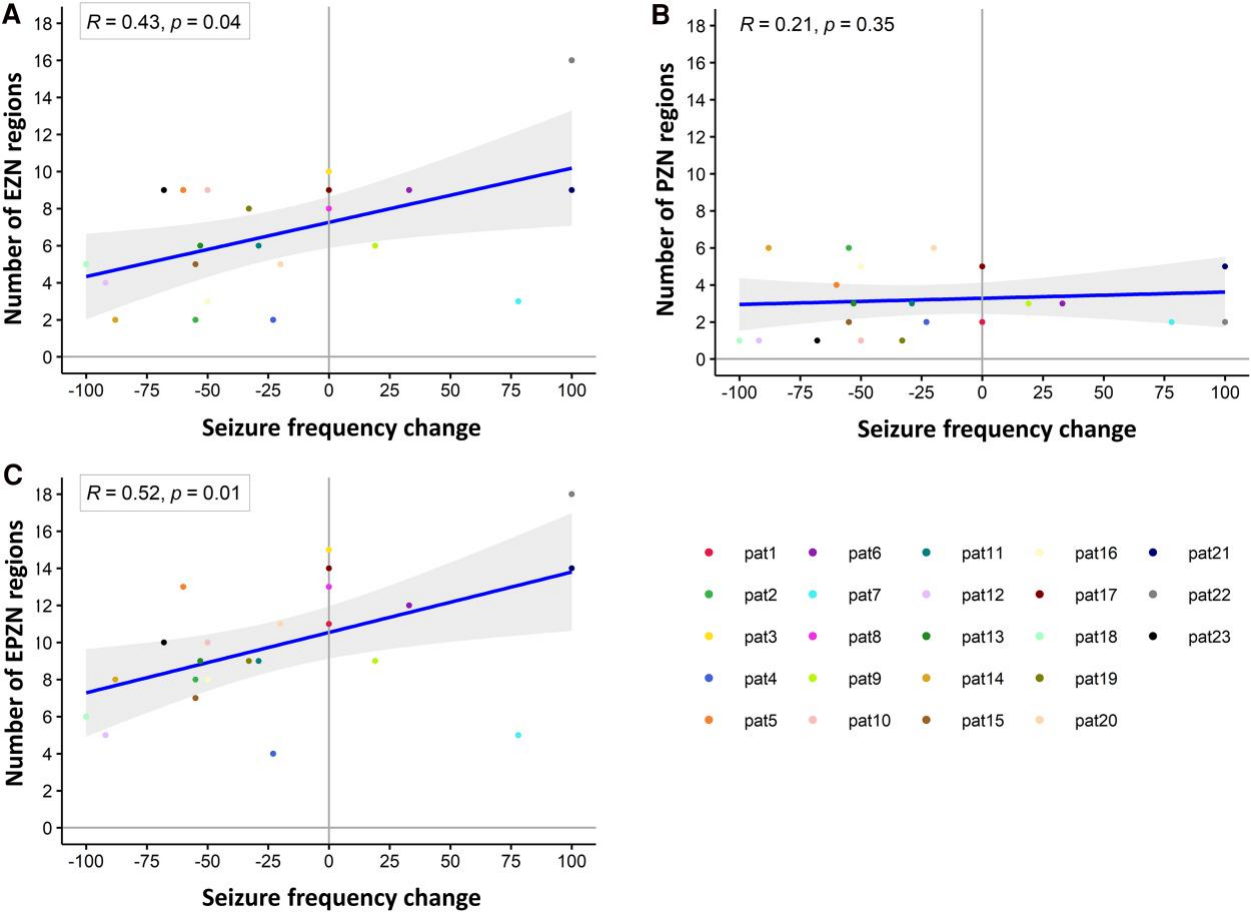
**Correlation between number of epileptogenic regions and changes in seizure frequency in patients with epilepsy after repeated sessions of tDCS. (A)** The scatter plot represents the regression of the number of EZN regions on the SF change after multiple cycles of multichannel tDCS in 23 patients with epilepsy. **(B)** Regression of the number of PZN regions on SF changes. **(C)** Correlation between the number of EPZN regions and SF changes.

In light of this trend, we further investigated the correlation between the SF change and the extent of epileptogenic regions. A significant positive correlation was observed between the SF changes and the extent of EZN regions (Spearman correlation *R* = 0.43, *P* = 0.04) (Fig. 4A). This suggested that when the EZN was smaller, the tDCS was more effective, resulting in a substantial reduction in SF. Conversely, with a larger EZN, patients tended to have no change or even an increase in the number of seizures compared with the baseline. No correlation was found between the change in SF and the extent of the PZN area (Fig. 4B). However, when the entire EPZN (comprising EZN and PZN) was taken into account, a significant positive correlation was observed between SF changes and the extent of the EPZN (Spearman correlation *R* = 0.52, *P* = 0.01; Fig. 4C).

Importantly, there was no statistically significant difference in the number of implanted regions between responders and non-responders (Wilcoxon test, *pval* = 0.69), and no significant correlation was observed between the total number of implanted regions and the number of EZN regions (Spearman correlation, *R* = 0.1, *pval* = 0.65).

We also estimated the extent of the EZN using volume rather than number of regions. The trends are similar, but the results were less significant (see Supplementary Figs 1 and 2).

### Effect of the depth of the EZN on tDCS response

Subsequently, we examined whether the mean distance to the scalp of the EZN could influence the clinical efficacy of multichannel tDCS. Following calculating EZN depth, we compared this measurement between the R and NR groups.

**Figure 5:**
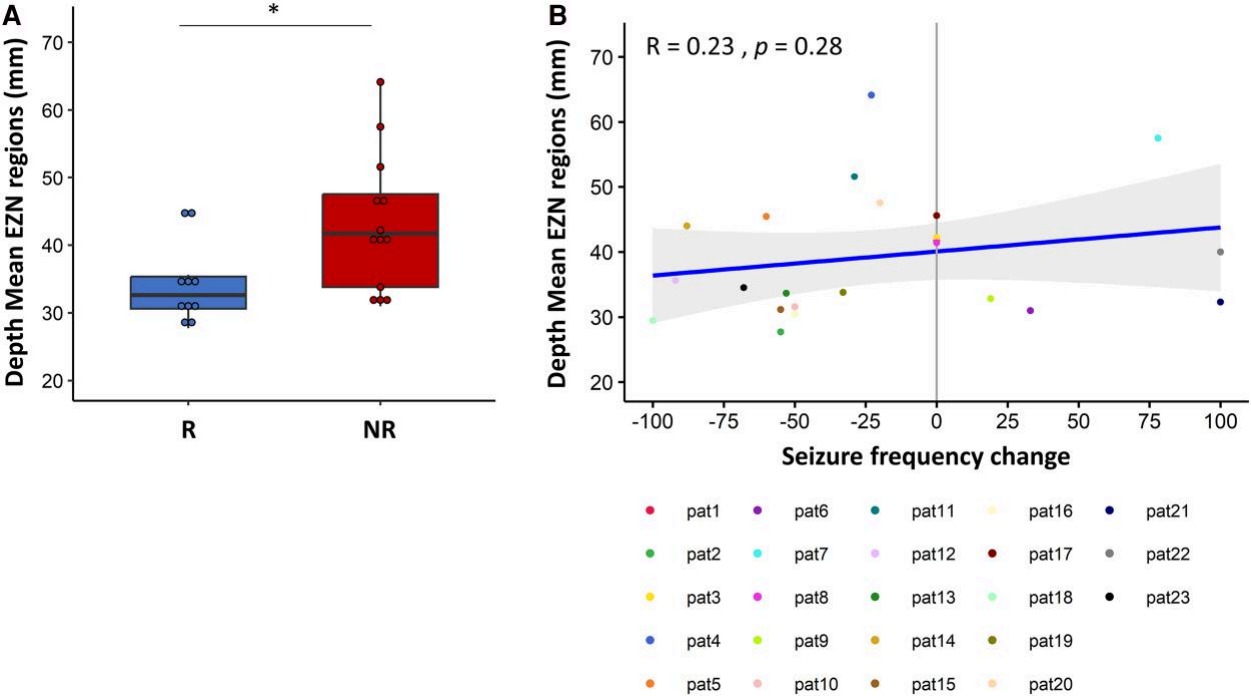
**Influence of EZ depth on clinical response to multichannel tDCS. (A)** Boxplots illustrate EZN depth determined by averaging the depth of each EZ regions (regions with normalized EI ≥ 0.4) for the responder (R) (In blue) and non-responder (NR) (in red) groups (*n* = 10 and *n* = 13, respectively). Each data point corresponds to the mean depth of EZ regions for each patient. The black bar with an asterisk marks a significant difference (*pval* = 0.025) between R and NR groups using the Wilcoxon test. **(B)** The scatter plot represents the Spearman correlation coefficient (R = 0.23) between the mean depth of EZ regions and the SF change after multiple cycles of multichannel tDCS.

We found that the EZN was significantly deeper in the NR group than in the R group (*W* = 29, *pval* = 0.025; Fig. 5A). This finding strongly suggests a potential association between the depth of the epileptogenic zone (targeted by our tDCS protocol) and the treatment response. The correlation analysis between the depth of EZN and the SF changes following tDCS cycles did not demonstrate statistical significance (Spearman correlation *R* = 0.23, *pval* = 0.28).

## Discussion

The main objective of this study was to explore how the extent of the epileptogenic network and the depth of the epileptogenic zone impact the clinical response to a multichannel tDCS protocol. The primary finding was a positive correlation between the extent of the epileptogenic network and the changes in seizure frequency after three tDCS cycles. Additionally, R and NR patients exhibited a significant difference in the mean depth of the epileptogenic zone.

### The clinical efficacy of tDCS is influenced by the extent of the epileptogenic network.

This retrospective analysis included 23 patients diagnosed with drug-resistant epilepsy. They underwent 3 weeks of daily stimulation over 6 months using a personalized multichannel protocol targeting epileptogenic zone.^16^ Among these patients, 10 were classified as ‘responders’ due to a reduction in SF of over 50% 2 months after the last stimulation compared with baseline. Nearly half of the patients experienced substantial clinical improvement (mean change −67% ± 19%). This ratio aligns with findings from other studies using tDCS in epilepsy.^3^ Our study observed a difference in the extent of the epileptogenic network (grouping the EZN and the PZN) between R and NR groups, which approached statistical significance. Specifically, the EPZN appeared more extended in NR than R. This finding was reinforced by the significant positive correlation between the extent of the EZN and EPZN and the changes in seizure frequency. In particular, when the epileptogenic regions were more focal, the tDCS was more efficient. Conversely, when the targeted areas for tDCS were extensive, this technique failed to reduce seizure occurrences. At the extreme, the broadest epileptogenic zones may lead to an increase in seizure frequency. The extension of epileptogenic networks is a proven limitation in surgical therapies, whether at the level of the EZN^39^ or PZN.^40^ Large-scale simulation studies show that it is more challenging to stop seizures when the network is more densely connected.^41^ These network parameters may be also limitations on the effectiveness of tDCS. These findings agree with a recent study^42^ that found that when the epileptogenic zone and whole network were widely extended across both hemispheres, cathodal tDCS demonstrated reduced therapeutic benefits. Finally, large epileptic networks may expose specific nodes to the risk of accidental excitation (anodes), thus exacerbating seizures. Therefore, it may be advisable to incorporate particular characteristics of the targeted anatomical zones to refine patient selection and potentially mitigate tDCS-related exacerbations observed in some NR patients. For instance, one could consider including the extent of the epileptogenic network in the criteria for patient inclusion.

### The effectiveness of tDCS in epilepsy is constrained by the depth of the epileptogenic zone network

Previous studies have highlighted the potential difficulty in reaching deep brain regions with low-intensity stimulation due to the distance between the electrodes and the targeted area.^19-22,43^ Our findings support this view, indicating a significant difference in the depth of the EZN in the brain in NR patients compared with R. These results diverge from computational studies developing epilepsy models to explore the spread of electric fields in the brain through various parameters of low-intensity stimulation application.^44^ Indeed, modeling studies have suggested that adjusting the number and positioning of scalp electrodes during tDCS could improve both the intensity and the focus of stimulation, even for deep brain targets.^8,20,45,46^ Human studies have corroborated these results using transcranial electrical stimulation during intracranial recordings.^43,47^ These latter studies proposed that transcranial electrical stimulation could induce an electric field capable of reaching deep structures like the hippocampus. Nevertheless, it is crucial to emphasize that the electric field detected in deep brain regions was relatively weak. Louviot *et al*.^47^ reported mean EF magnitudes of 0.21, 0.17, and 0.07 V.m^−1^ in the amygdala, hippocampus, and cingulate gyrus, respectively, in a study combining SEEG recordings with transcranial electrical stimulation. Our group recently found a remarkably low magnitude of effects on deep brain structures in epileptic patients who underwent simultaneous SEEG recording.^43^ To date, none of these studies have been clinically validated. These rare investigations involved a relatively small number of patients, reflecting the inherent challenges in conducting protocols with transcranial stimulation and intracranial recordings. Notably, the potential clinical benefits of tDCS, such as reduced seizure frequency, are believed to arise from both immediate and plasticity effects (for a more comprehensive review, see Simula *et al*.^3^ and Liu *et al*.^48^). These complex mechanisms involve functional changes within brain networks, which could be crucial for mitigating epileptic activity.^13,14,16,49^ However, our results suggest that targeting deep brain regions with tDCS can be challenging. This difficulty arises from the reduced strength of the electrical field as the distance from the scalp increases and from the inability to control the direction of the electric field when targeting deep brain areas. New stimulation paradigms could be of interest in this context. A novel stimulation technique, known as temporal interference, has proven its ability to reach and stimulate deep brain regions by generating interferences at points located distant from the surface electrodes.^50^ A recent investigation conducted on cadavers revealed superior penetration of temporal interference fields into the human hippocampus compared with transcranial current stimulation without affecting the surrounding tissues.^51^

### Limitations

While our study sheds light on the potential efficacy of tDCS in managing epilepsy, highlighting in particular the crucial role of the extent and depth of the epileptogenic zone network, several limitations should be acknowledged. The main limitation of this study stems from the heterogeneity in SEEG implantation among patients. The SEEG electrode placement was tailored based on individual non-invasive assessments, leading in variations in electrode positioning and brain coverage. This variability may have restricted our ability to fully characterize the epileptogenic network and its relationship with tDCS efficacy. Additionally, the method of labeling entire brain regions as epileptogenic based on limited SEEG contacts with high epileptogenicity may oversimplify the complexity of the network.

To estimate the extent of the epileptogenic zone (EZN), we counted the number of virtual epileptic patient (VEP) atlas parcellations containing SEEG contacts with high epileptogenicity index (EI) values. This approach was chosen because the VEP atlas is specifically designed to reflect functional regions crucial for describing the organization of epileptogenic networks. While this method provides a broad overview of the network, it may not capture all specific areas with high epileptogenicity, potentially impacting the precision of our findings.

Another limitation of our study is the use of patient-reported seizure diaries, estimated to have <50% accuracy.^52^ This under-reporting introduces potential variability and bias in the data. Future studies should integrate automatic seizure monitoring methods to improve accuracy and reliability.

To conclude, the retrospective nature of this study and the relatively small number of subjects included are limitations of our study. However, we can show that both the extension of epileptogenic networks and their depth relative to the surface are factors limiting the effectiveness of tDCS. This underscores the need to explore alternative stimulation methods and/or optimize the current process.

## Supplementary Material

fcaf012_Supplementary_Data

## Data Availability

The data that support the findings of this study are available on request from the corresponding author. The data are not publicly available due to clinical restrictions. the codes generated and used in this work are available through a repository (https://gitlab-dynamap.timone.univ-amu.fr/epileptogenic-zone-characteristics-determine-effectiveness-of-electrical-transcranial-stimulation-in-epilepsy-treatment/dev).
