# Supplementary material for: Epileptogenic zone characteristics determine effectiveness of electrical transcranial stimulation in epilepsy treatment
Source: Brain Commun. 2025 Feb 19;7(1):fcaf012. doi: 10.1093/braincomms/fcaf012 (PMC11837341; doi:10.1093/braincomms/fcaf012)

**Supplementary Tables**

Supplementary Table 1. Summary of stimulation electrodes, electrode names, and delivered intensities per patient.

| Patient | Number of tDCS electrodes | Cathodal electrodes and intensity (µA) | | Anodal electrodes and intensity (µA) | | Total Injected Current (µA) |
| --- | --- | --- | --- | --- | --- | --- |
| PAT 1 | 8 | CP6  P4  T8 | -248  -506  -1128 | F7  P7  PO7  T7  Cz | 369  509  401  403  200 | 1884 |
| PAT 2 | 6 | P4  P5  TP8 | -872  -699  -428 | C3  P9  T7 | 1374  348  277 | 1999 |
| PAT 3 | 7 | CP5  F7  T7 | -539  -767  -690 | CP6  F8  P8  Cz | 499  642  655  200 | 1996 |
| PAT 4 | 7 | F8  FP1  TP7 | -484  -478  -1036 | C3  FC3  FC5  P3 | 200  273  930  595 | 1998 |
| PAT 5 | 7 | F7  P7  T7 | -508  -1220  -270 | F8  P4  P8  T8 | 394  245  948  411 | 1998 |
| PAT 6 | 8 | C2  P8  Pz  T8  Cz | -413  -240  -235  -332  -777 | AF7  F4  O1 | 377  867  753 | 1997 |
| PAT 7 | 7 | AF7  F8  FP1  T7 | -1346  -237  -215  -200 | AF3  C3  F3 | 521  489  988 | 1998 |
| PAT 8 | 8 | F7  P3  P7  T7 | -380  -508  -910  -200 | C3  Oz  T8  Cz | 885  713  200  200 | 1998 |
| PAT 9 | 6 | C1  C2  CP2 | -1393  -352  -253 | F6  FC6  P8 | 396  687  915 | 1998 |
| PAT 10 | 7 | C1  FC1  P3  Pz | -563  -571  -487  -378 | FC6  FP1  P8 | 726  464  809 | 1999 |
| PAT 11 | 7 | CP5  F8  P7  P8  T7 | -627  -552  -224  -311  -284 | CP1  P3 | 807  1191 | 1998 |
| PAT 12 | 7 | FC1  FC2  F4 | -1184  -200  -613 | F8  AF7  CP5  CP6 | 704  537  445  311 | 1997 |
| PAT 13 | 8 | C4  CPz  FC6  Cz | -314  -1158  -220  -306 | AF4  F2  P6  TP8 | 349  482  967  200 | 1998 |
| PAT 14 | 8 | CP1  Cz | -703  -1295 | CP5  F6  FP2  P3  P6  TP8 | 207  281  402  376  417  315 | 1998 |
| PAT 15 | 7 | AF3  C3  CP3  F3 | -200  -713  -447  -639 | CP2  F8  Iz | 547  732  720 | 1999 |
| PAT 16 | 4 | C6  Cz | -330  -1669 | F2  P4 | 1118  881 | 1999 |
| PAT 17 | 7 | AF7  C4  P7 | -569  -801  -627 | C1  CP6  FC6  PO8 | 829  368  588  212 | 1997 |
| PAT 18 | 7 | C2  C3  CP2 | -584  -686  -728 | F1  F3  P5  T7 | 360  350  596  692 | 1998 |
| PAT 19 | 8 | C2  C3  Cz | -714  -491  -794 | F1  F3  FC5  P5  TP7 | 720  200  557  293  229 | 1999 |
| PAT 20 | 6 | AF7  F7  P9  T7 | -376  -454  -355  -814 | C3  FC3 | 1548  451 | 1999 |
| PAT 21 | 8 | C2  C4  C6  CP2 | -200  -548  -499  -752 | F6  P4  P8  Cz | 350  349  438  862 | 1999 |
| PAT 22 | 8 | F5  P10  T7  TP7 | -585  -720  -252  -442 | F7  FC1  P5  Cz | 329  515  955  200 | 1999 |
| PAT 23 | 8 | C2  C3  FC5  Cz | -221  -295  -576  -907 | F3  FP1  P3  T7 | 500  299  508  692 | 1999 |

Supplementary Table 2. Comprehensive listing of EZ and PZ regions for each patient.

| **Patients** | **Epileptogenic hemisphere** | **EZ regions** | **PZ regions** |
| --- | --- | --- | --- |
| *Pat 1* | Right hemisphere | T1 planum temporale,  Parietal operculum, STS anterior, Thalamus, Insula gyri longi, Central sulcus upper limb, Caudate nucleus, F3 pars opercularis, Central operculum | Gyrus of Heschl, Putamen |
| *Pat 2* | Left hemisphere | Cuneus, O2 | Hippocampus-posterior, O1, STS posterior, Lingual sulcus, Collateral sulcus, Precuneus |
| *Pat 3* | Bilateral | Left T1 lateral posterior, Left insula gyri longi, Left STS anterior, Left hippocampus posterior, Left STS posterior, Left T2 posterior, Left Collateral sulcus, Left temporal pole, Left T3 posterior, Left lingual gyrus | Right parahippocampal cortex, Right rhinal cortex,  Left parahippocampal cortex, Left O2, Left calcarine sulcus |
| *Pat 4* | Left hemisphere | Parahippocampal cortex, Collateral sulcus | Insula gyri brevi, F3 pars opercularis |
| *Pat 5* | Left hemisphere | Hippocampus anterior, Rhinal cortex, Hippocampus posterior, Occipito-temporal sulcus, T1 planum temporale, Gyrus of Heschl, T1 lateral posterior, STS posterior, Calcarine sulcus | T3 posterior, T2 posterior, Insula gyri longi, T1 lateral anterior |
| *Pat 6* | Right hemisphere | STS posterior, Intraparietal sulcus, Supramarginal posterior, Angular gyrus, T3 posterior, T1 planum temporale, Gyrus of Heschl, STS anterior, T1 lateral posterior | Postcentral sulcus, Intraparietal sulcus, Occipito-temporal sulcus |
| *Pat 7* | Left hemisphere | Amygdala, Hippocampus anterior, Fusiform-gyrus | F2 rostral, Orbito-frontal cortex |
| *Pat 8* | Bilateral | Left temporal pole, Left T1 planum polare, Left T2 anterior, Left parahippocampal cortex, Left Insula gyri brevi, Left T2 posterior, Left STS posterior, Left Orbito-frontal cortex | Left ITS anterior, Left T3 posterior, Left F2 caudal Right orbito-frontal cortex, Left STS anterior |
| *Pat 9* | Right hemisphere | Intraparietal sulcus, Superior parietal lobule P1, Middle cingulate cortex posterior,  Central sulcus upper limb, Precentral gyrus upper limb,  Supramarginal posterior | Middle frontal sulcus, Postcentral sulcus, Anterior-cingulate cortex |
| *Pat 10* | Bilateral | Right-SMA, Left-SMA, Left SFS caudal, Left paracentral lobule, Left central sulcus upper limb, Left superior parietal lobule P1, Left O1, Left O2, Right STS posterior | Left intraparietal sulcus |
| *Pat 11* | Left hemisphere | Thalamus, Ventral DC, Rhinal cortex, Temporal pole, Parietal operculum, T3 anterior | Insula gyri brevi, STS anterior, Intraparietal sulcus |
| *Pat 12* | Right hemisphere | Precentral sulcus inferior part, Middle cingulate cortex posterior, Paracentral lobule,  Postcentral gyrus | F2 caudal |
| *Pat 13* | Bilateral | Right SFS caudal, Right F2 caudal, Right SMA, Right precentral sulcus superior, Right paracentral lobule, Right insula gyri brevi | Right postcentral gyrus, Right anterior cingulate cortex, Left Middle cingulate cortex anterior |
| *Pat 14* | Left hemisphere | SMA, Marginal branch of the cingulate sulcus | Middle cingulate cortex posterior, Central sulcus upper limb, Precentral gyrus upper limb, Precuneus, Paracentral lobule, Posterior cingulate cortex dorsal |
| *Pat 15* | Left hemisphere | Postcentral sulcus, Supramarginal posterior, Central sulcus head face, Precentral gyrus upper limb, Precentral sulcus inferior | F2 caudal, Central sulcus upper limb |
| *Pat 16* | Right hemisphere | Paracentral lobule, Central-sulcus upper limb, Precentral gyrus upper limb | Insula gyri brevi, F3 pars opercularis, Parietal operculum, Parieto-occipital sulcus, Precentral sulcus superior |
| *Pat 17* | Left hemisphere | Temporal pole, Hippocampus anterior, Hippocampus posterior, Occipito-temporal sulcus, Middle frontal sulcus, Collateral sulcus, Fusiform gyrus, T3 posterior, T1 lateral anterior | Amygdala, T2 anterior, T3 anterior, STS anterior, Orbito-frontal cortex |
| *Pat 18* | Left hemisphere | Precentral gyrus upper limb, Precentral gyrus head face, Central sulcus upper limb, Postcentral gyrus, Precentral sulcus superior | Middle cingulate cortex posterior |
| *Pat 19* | Left hemisphere | Paracentral lobule, F1 lateral premotor, Precentral gyrus upper limb, Central sulcus upper limb, Postcentral gyrus, Marginal branch of the cingulate sulcus, Postcentral sulcus, SMA | Central sulcus head face |
| *Pat 20* | Bilateral | Left temporal pole, Left T1 planum polare, Left ITS anterior, Left amygdala, Left insula gyri longi | Left T1 lateral anterior, Left rhinal cortex, Left T3 anterior, Left hippocampus posterior, Left insula gyri brevi, Right rhinal cortex |
| *Pat 21* | Right hemisphere | Precentral sulcus superior, Central sulcus upper limb, Central sulcus head face, Postcentral gyrus, T1 planum temporal, Supramarginal anterior, Intraparietal sulcus, Marginal branch of the cingulate sulcus, Superior parietal lobule P1 | Precentral gyrus upper limb, Middle cingulate cortex posterior, Parietal operculum, Paracentral-lobule, Postcentral-sulcus |
| *Pat 22* | Left hemisphere | STS anterior, T2-anterior, Amygdala, Hippocampus anterior, ITS anterior, ITS-posterior, T2-posterior, Gyrus of Heschl, T1 lateral posterior, Insula gyri longi, Insula gyri brevi, Inferior frontal sulcus, Left F3, pars-triangularis, T3-anterior, Rhinal-cortex, Temporal-pole | Orbito-frontal cortex, T1 planum polare |
| *Pat 23* | Left hemisphere | Middle cingulate cortex posterior, SFS caudal, Precentral sulcus superior, Central sulcus upper limb, Precentral gyrus upper limb, Postcentral gyrus, Insula gyri brevi, Precentral gyrus head face, Paracentral lobule | Postcentral sulcus |

Supplementary Table 3: Baseline seizure counts for all patients. Estimated seizure count per month at baseline, calculated by averaging the seizure counts over two months.

| Patient | Seizure count per month at baseline |
| --- | --- |
| PAT 1 | 35 |
| PAT 2 | 66.5 |
| PAT 3 | 4 |
| PAT 4 | 9.2 |
| PAT 5 | 20 |
| PAT 6 | 26 |
| PAT 7 | 2.5 |
| PAT 8 | 4 |
| PAT 9 | 10.07 |
| PAT 10 | 6 |
| PAT 11 | 7.5 |
| PAT 12 | 300 |
| PAT 13 | 51.5 |
| PAT 14 | 4 |
| PAT 15 | 4.4 |
| PAT 16 | 108.4 |
| PAT 17 | 10 |
| PAT 18 | 73.5 |
| PAT 19 | 101.1 |
| PAT 20 | 4 |
| PAT 21 | 18 |
| PAT 22 | 9.67 |
| PAT 23 | 22 |

Supplementary Table 4. Summary of implanted regions: Counts of EZN and PZN per patient.

| Patient | Total number of implanted regions | Number of EZN regions | Number of PZN regions |
| --- | --- | --- | --- |
| PAT 1 | 23 | 9 | 2 |
| PAT 2 | 32 | 2 | 6 |
| PAT 3 | 20 | 10 | 5 |
| PAT 4 | 43 | 2 | 2 |
| PAT 5 | 40 | 9 | 4 |
| PAT 6 | 25 | 9 | 3 |
| PAT 7 | 20 | 3 | 2 |
| PAT 8 | 28 | 8 | 5 |
| PAT 9 | 13 | 6 | 3 |
| PAT 10 | 32 | 9 | 1 |
| PAT 11 | 42 | 6 | 3 |
| PAT 12 | 23 | 4 | 1 |
| PAT 13 | 37 | 6 | 3 |
| PAT 14 | 19 | 2 | 6 |
| PAT 15 | 34 | 5 | 2 |
| PAT 16 | 25 | 3 | 5 |
| PAT 17 | 26 | 9 | 5 |
| PAT 18 | 10 | 5 | 1 |
| PAT 19 | 14 | 8 | 1 |
| PAT 20 | 33 | 5 | 6 |
| PAT 21 | 43 | 9 | 5 |
| PAT 22 | 41 | 16 | 2 |
| PAT 23 | 20 | 9 | 1 |

**Supplementary Figures**

**Supplementary Figure 1. Comparing the volume of epileptogenic regions in responder (R) and non-responder (NR) patients.** Boxplots illustrate EZN, PZN and EPZN extent determined by the total volume of EZN, PZN and EPZN regions for responders (in green) and non-responders (in red) groups (respectively n=10 and n=13). Each data point corresponds to the total volume of the specific brain regions considered for each patient (EZN, PZN and both).


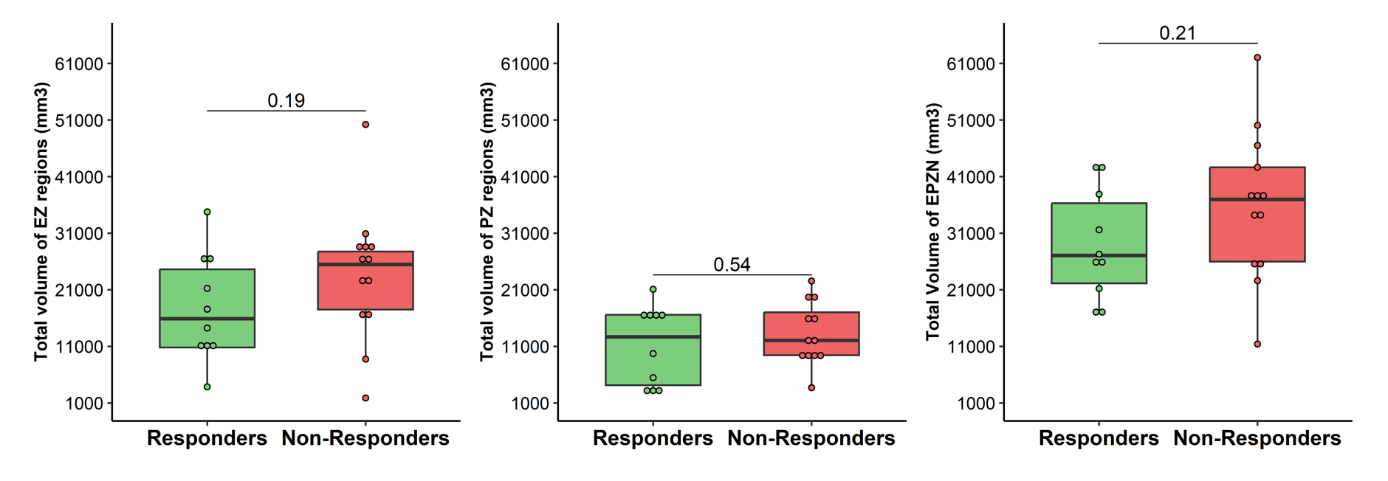


**Supplementary Figure 2. Correlation between volume of epileptogenic regions and changes in seizure frequency in patients with epilepsy after repeated sessions of tDCS.** The scatter plot represents the regression of the number of EZN PZN and EPZN regions on the SF change after multiple cycles of multichannel tDCS in 23 patients with epilepsy.


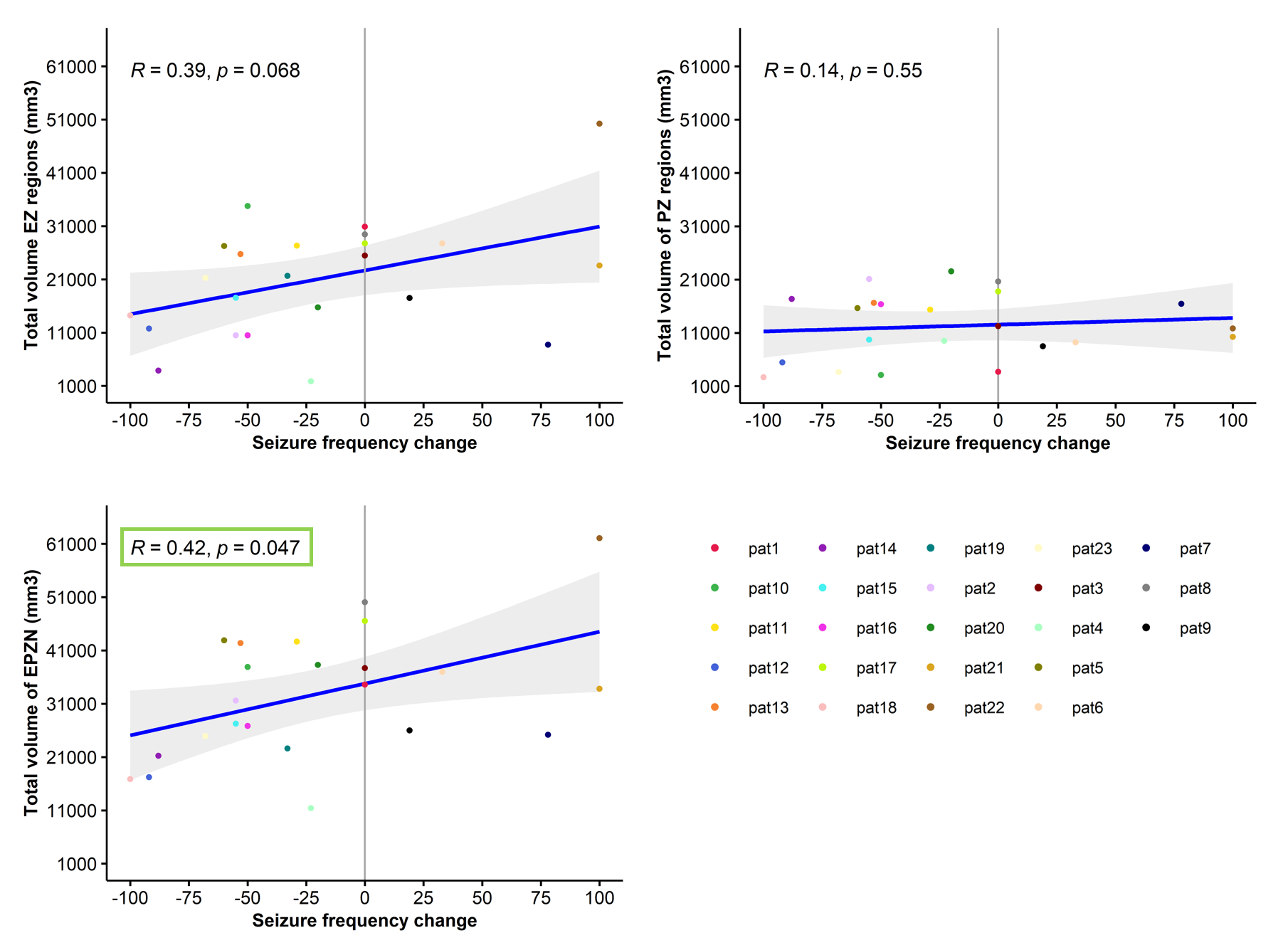

Supplement: fcaf012_Supplementary_Data [file fcaf012_supplementary_data.docx]
